# Diversity Assessment and DNA-Based Fingerprinting of Sicilian Hazelnut (*Corylus avellana* L.) Germplasm

**DOI:** 10.3390/plants11050631

**Published:** 2022-02-25

**Authors:** Maria Carola Fiore, Annalisa Marchese, Antonio Mauceri, Ignazio Digangi, Anna Scialabba

**Affiliations:** 1 Council for Agricultural Research and Economics—Research Centre for Plant Protection and Certification, S.S. 113 km 245,500, 90011 Bagheria, Italy; 2 Department of Agricultural, Food and Forest Sciences, University of Palermo, Viale delle Scienze—Ed. 4, 90128 Palermo, Italy; annalisa.marchese@unipa.it; 3 Department Agraria, University Mediterranea of Reggio Calabria, Loc. Feo di Vito snc, 89065 Reggio Calabria, Italy; antonio.mauceri87@unirc.it; 4 Living Plants Germplasm Bank of Nebrodi, Contrada Pirato, 98060 Ucria, Italy; ignazio.digangi62@gmail.com; 5 Department of Biological, Chemical and Pharmaceutical Science and Technologies (STEBICEF), University of Palermo, Via Archirafi 38, 90123 Palermo, Italy; anna.scialabba@unipa.it

**Keywords:** *Corylus avellana* L., germplasm conservation, genetic diversity, microsatellites, DNA fingerprint

## Abstract

The characterization of plant genetic resources is a precondition for genetic improvement and germplasm management. The increasing use of molecular markers for DNA-based genotype signature is crucial for variety identification and traceability in the food supply chain. We collected 75 Sicilian hazelnut accessions from private and public field collections, including widely grown varieties from the Nebrodi Mountains in north east Sicily (Italy). The germplasm was fingerprinted through nine standardized microsatellites (SSR) for hazelnut identification to evaluate the genetic diversity of the collected accessions, validating SSR discrimination power. We identified cases of homonymy and synonymy among acquisitions and the unique profiles. The genetic relationships illustrated by hierarchical clustering, structure, and discriminant analyses revealed a clear distinction between local and commercial varieties. The comparative genetic analysis also showed that the Nebrodi genotypes are significantly different from the Northern Italian, Iberian, and Turkish genotypes. These results highlight the need and urgency to preserve Nebrodi germplasm as a useful and valuable source for traits of interest employable for breeding. Our study demonstrates the usefulness of molecular marker analysis to select a reference germplasm collection of Sicilian hazelnut varieties and to implement certified plants’ production in the supply chain.

## 1. Introduction

*Corylus avellana* L. is probably native from Asia Minor due to its wide distribution and also as wild forms in Pontus (an ancient province in northern Anatolia). This theory is also supported by the name of hazelnut fruits, Pontikón (káryon) or (karua) Pontika, káryon Pontikón (Ponto nut) given by Greeks [1]. The most common name is “hazelnut”, while the less known is ‘filberts’, which refers to the long leafy husks covering the nut of some hazelnut varieties [2]. It is a diploid (2n = 2x = 22) monoecious, dichogamous, and wind-pollinated species [3,4], which also presents sporophytic self-incompatibility [5,6] promoting out-crossing. Therefore, many varieties are highly heterozygous and clonally propagated [4].

Hazelnut trees grow wild in many regions of the world, through Europe, Asia, and North America, but the longest history of human cultivation belongs to Europe [7]. The important role of hazelnut as a plant resource in the food economics has been deduced in archaeological Mesolithic sites [8,9]. It is not yet certain when and where the hazelnut domestication began. The results by Boccacci and Botta [7] sustained two separate domestication events, in the western and eastern Mediterranean basin. The first certain evidence of specialized hazelnut cultivation refers to the presence of fruit in cemeteries [10] and some details of the Italian farming practices reported in ancient Greek and Roman writings (300–200 BC) [11].

Hazelnut cultivation areas are continuously growing around the world, with an average cultivated area of 971,751 ha and a yield of 818,172 tons/year in 2016–2020 [12]. During the same period, Italy ranked as the second-largest producing country in the world (average 124,729 tons), after Turkey (average 610,209 tons) and ahead of the USA (average 44,816 tons), Iran (average 13,677 tons), France (average 121,512 tons), and Spain (average 9169 tons), followed by Chile, Poland, Serbia, Kyrgyzstan, and Uzbekistan [12]. Nowadays, in Italy, the average hazelnut area harvested is 76,255 ha, according to the Italian National Institute of Statistics (ISTAT) [13]. Latium recorded the highest average value of hazelnut harvested production (39,348 tons) among the Italian regions, followed by Campania (37,855 tons), Piedmont (34,991 tons), and Sicily (16,174 tons) [13], the latter together with Campania having the highest number of cultivated varieties.

Under Greek rule, hazelnut was introduced in Sicily and spread in the Madonie and Nebrodi Mountains under arid conditions, at altitudes ranging from 180 to 1150 m above sea level [14]. Hazelnut plantations began to expand in this territory in 1890 due to the high productivity, adaptability, and ability to protect the soil from hydrogeological instability [15,16,17].

Starting from the 1960s, the traditional hazelnut cultivation in the Nebrodi area underwent a slow and steady decline that has led to the genetic erosion of many cultivars and the persistence of fewer specimens of others. Until now, 10,500 ha are still cultivated in the Nebrodi Mountains [13] due to their favorable climatic and ecological conditions and the valuable hazelnut production, particularly appreciated for their persistent aroma and flavor, making them very suitable and widely used in local artisan pastry. The main local varieties currently cultivated in Sicily are ‘Carrello’, ‘Curcia’, ‘Pannottara’, ‘Ghirara’ also known by the local name ‘Agghirara’ [14], several ‘Minnulara’ genotypes, Sicilian ecotypes of ‘Nocchione’, and other ancient varieties. Many of these varieties are characterized by high hardiness and capacity to survive in harsh environmental conditions [14,18]. Some of these varieties are currently registered in the Italian National Register of Plant Variety, where several synonymies/homonymies are identifiable. 

The lack of innovative cropping management practices in Sicilian hazelnut orchards, due to unfavorable orographic conditions, and the high variability of local varieties result in lower crop yield per unit areas (on average 1 t/ha) compared to an average production of 1.8 t/ha registered in other Italian regions (Piedmont, Latium, and Campania) [13]. Local varieties have probably remained unaltered for hundreds of years, influencing the genetic variability and its distribution. These varieties have been grown in restricted mountain territories, arising the spread of locally adapted clones whose identification is uncertain or based on morphological traits of nuts, husks, and other descriptors [18,19,20]. Moreover, variety identification is difficult because vegetative propagation is achieved before the distinguishing traits are developed [21,22]. DNA genotyping can identify synonymies/homonymies and molecular variants within germplasm collections. Sustainable crop production together with the enhancement of autochthonous cultivars and a certified production system (from nursey to market) are the strategic goals to support the relaunch of the Sicilian hazelnut food chain. 

Cultivar and clone identification are some of the most important aspects of modern cropping systems [23]. The traceability implementation in the food supply chain represents a crucial point for increasing quality and safety, optimizing production, and documenting its sustainability.

The DNA-based molecular markers are excellent tools for exploring the genetic diversity in plants. In particular, single sequence repeats (SSRs) or microsatellites have long been the preferred class of molecular markers for genotyping vegetatively propagated crops for their ability to discriminate at any stage of development, based on SSR multiallelism, high polymorphism, cost-effectiveness, and high reproducibility [24]. SSRs have been proven to be highly effective tools in assessing genetic diversity also in *Corylus* *avellana* L. [25,26,27,28,29,30,31,32,33]. By these molecular markers, hazelnut germplasm collections have been fingerprinted, defining synonymies and homonymies [34,35,36], assessing genetic relationships and parentage [37,38,39,40,41], investigating the genetic structure of different populations comparing local cultivars and wild genotypes [29,32,38,42,43], and developing genetic maps for association analysis [44,45]. More recently, single-nucleotide polymorphisms (SNPs) based on next-generation sequencing (NGS) have been utilized in hazelnut linkage maps [46,47] to assess the genetic diversity and to investigate the domestication history of European hazelnut [48,49,50]. Although much progress has been made with a significant reduction in SNP genotyping cost, SSR markers still represent a valuable, cost-effective, transferable, and extensively used tool to ascertain the identity of the accessions, to solve cases of homonymy and synonymy in hazelnut genetic resources. 

The main commercial and local Sicilian hazelnut varieties are currently present in ex situ collections, both private germplasm collections and in the public Living Plant Germplasm Bank (LPGB) of Ucria (Messina, Italy) established within the Nebrodi Regional Park. The LPGB has carried out research activities devoted to the conservation and exploitation of local plant genetic resources together with the Sicilian Plant Germplasm Repository at the University of Palermo for several years. 

The present study aimed to fingerprint by SSRs hazelnut landraces, local and commercial varieties, commonly cultivated in the Nebrodi Mountains to verify their identity and relationships. Genetic analysis will allow for identifying synonyms and homonyms between accessions for better management of the germplasm collections and the certification of hazelnut propagation material.

## 2. Results

### 2.1. Evaluation of DNA Extraction Methods

Comparison of DNA extraction efficiency of three methodologies produced different levels in terms of yield, purity, and DNA degradation over time. All three methods were not comparable for DNA yield varying requirements in terms of purity and DNA degradation level (Table S1). 

The method by Doyle and Doyle (1987), without beta-mercaptoethanol addition, produced a high DNA yield with a low storage stability after ten days at −20 °C, affecting PCR performance due to a putative DNA degradation or presence of amplification DNA inhibitors. By contrast, the commercial kit NucleoSpin^®^ Plant II (Macherey-Nagel) produced low DNA yield with low A260/230 ratios, due to phenolics and polysaccharides contamination. Moreover, the A260/280 ratios did not result optimal in most of the samples, causing a low PCR performance and quick degradation of DNA over time (after 3 days), invalidating the following amplification analysis (data not show). Martínez-González et al.’s (2017) CTAB-based modified protocol provided better results for DNA integrity, purity, concentration, and storage stability. The modification applied allowed us to obtain pure and high-quality DNA suitable for further molecular analysis. In particular, good storage stability was reached (up to 12 months at −20 °C), the 260/280 purity ratio ranged between 1.85 and 1.9, and the 260/230 ratio ranged between 2.0 and 2.08 for all extracted samples. Ten samples were randomly selected to check the purity of gDNA by individual and multiplex PCR amplification using three different SSRs. 

### 2.2. Genetic Diversity

The genetic uniqueness of locus profiles was analyzed on allelic profiles of 75 hazelnut accessions sampled on the Nebrodi Mountains of Sicily (Table S2). Multi-locus match analysis identified 13 unique profiles, including 14 as contracted multi-locus genotypes. The accessions were considered as duplicates when they had an identical SSR profile or allelic difference with a few base pairs in one SSR locus, considering that some genotyping errors and/or spontaneous SSR mutations could occur. In detail, multi-locus match analysis identified two commercial varieties, thirteen local varieties, three landraces, and twelve unknown genotypes (LPGB field collection). One profile (n.26) grouped 15 LPGB local accessions, the accessions FC4_10 and FC4_11, indicated as ‘Minnulara Don Ciccio’ by the farmer during sampling, and the accessions identified as ‘Nostrale Mollese’ and ‘Santa Maria di Gesù’ (FC4_09) (Table S3). Two accessions named ‘Parrinara’ were included in two different profiles due to a difference of three alleles in three different loci (CAT-B502, CAT-B504, and CAT-B505). One sample (FC4_07), incorrectly identified as ‘Ghirara’ by the farmer, showed a unique profile (n.30) and was renamed ‘Baratta01’, considering it as landraces from this point on in further genetic analysis.

To verify the correspondence of the thirteen unique profiles with those of Italian germplasm previously investigated, a match analysis was performed to determine all cases of identity and synonyms, presumably corresponding to clonal genotypes with the indeterminate presence of replicated clonal mutations. The match analysis was conducted on allelic profiles obtained from the eight loci in common (CATB105 was excluded). The accession FC4_08 and the variety ‘Tonda Gentile delle Langhe’ (field collection sample—University of Turin) showed a unique profile and therefore, FC4_09 was no longer considered as a Sicilian landrace. It was not possible to discriminate between the two ‘Parrinara’ accessions or assign the correct profile due to the lack of published reference data. To avoid bias in genetic analyses, the redundant accession included in 13 genetic profiles were further removed from the dataset before assessing genetic diversity parameters, as reported in Table S4.

A genotype accumulation curve was calculated to assess the minimum number of loci necessary to discriminate between unique genotypes, considering an increasing number of SSR markers. The curve reached a plateau at eight loci (Figure S1), indicating that the present set of SSRs is satisfactory and statistically relevant to identify the varieties included in this study.

The nine polymorphic SSR markers detected a total of 69 alleles with an average of 7.67 alleles per marker (Table 1). The number of observed alleles (Na) ranged from 4 (CAT-B505) to 10 (CATB504 and CAT-B507). According to the results, the polymorphism information content (PIC) ranges from 0.332 (CAT-B105) to 0.659 (CAT-B504), with an average of 0.518. Eight SSR loci were highly polymorphic (PIC > 0.5) and one (CAT-B105) was moderately polymorphic (0.5 < PIC > 0.25). The observed heterozygosity values (Ho) ranged from 0.367 (CATB105) to 1.0 (CATB505). The levels of expected heterozygosity (He) ranged from 0.347 (CAT-B105) to 0.760 (CAT-B504) with an average of 0.670. The discriminating power (D) of the nine SSR markers ranged between 0.328 (CAT-B105) and 0.659 (CAT-B504). The unbiased expected heterozygosity (uHe) ranged from 0.353 (CAT-B105) to 0.774 (CAT-B 504), with an average value of 0.684. In general, the D values echo those obtained for He and PIC except for the CAT-B502 locus, which with the lowest PD value (0.267) registered a PIC value (0.638) very close to the average value.

**Figure 1:**
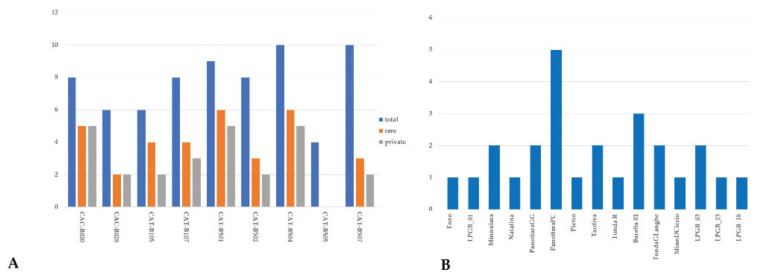
Distribution of rare and private alleles in each locus (**A**) and cultivars with private alleles (**B**) detected on 30 hazelnut genotypes sampled in the Nebrodi Mountains.

The allelic frequencies made it possible to observe the allele distribution and to identify rare and private alleles (Figure 1, Table S5). Sicilian hazelnut germplasm showed the presence of 34 rare alleles through all loci, representing 49.3% of total allele diversity, of which 25 were private alleles (allele present only in one accession). A higher number of rare alleles (six) was observed at locus CAT-B501 and locus CAT-B504. Rare alleles were not observed at locus CAT-B505, and only one allele (277) at locus CAC-B020 was present in homozygous state (‘Pietro’) (Figure 1A and Table S5). In total, seven local varieties, two commercial varieties, two landraces, and four LPGB genotypes had private alleles (Figure 1B). Five local varieties, currently cultivated in Nebrodi Mountains, ‘Curcia’, ‘Ghirara’, ‘Rossa Galvagno’, ‘Minnulara Rocco’, and ‘Carrello’, and the two accessions of ‘Parrinara’ did not show rare alleles. Among local varieties, ‘Panottara Piano Campo’ showed the higher number (five) of rare alleles, of which four were private. Genotype ‘Baratta01’ had the highest number of private alleles (three) among local genotypes. All three pollinizer genotypes (‘Tardiva’, ‘Minnulara’, and ‘Natalina’) showed the presence of private alleles at four (CAC-B020, CAC-B105, CAT-B501, and CAT-B507) of the nine SSR loci (Table S5).

**Figure 2:**
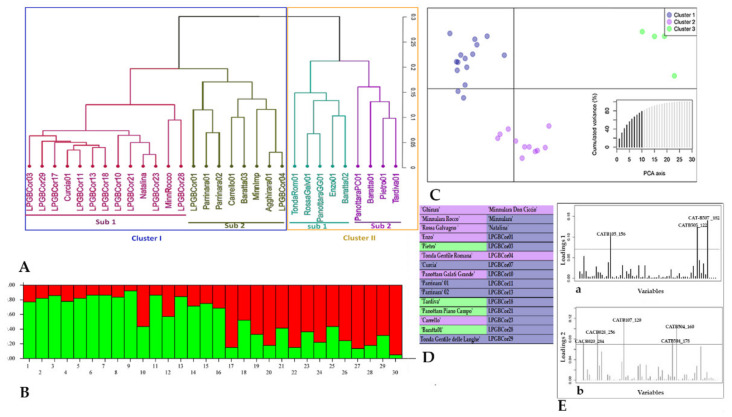
(**A**) UPGMA dendrogram based on Bruvo’s distance coefficient. (**B**) STRUCTURE analysis of 30 Sicilian hazelnut genotypes, using K-values for two groups. STRUCTURE plot showing the proportion of inferred ancestry (Q) in the genetic clusters identified within 30 genotypes. (**C**) Discriminant analysis of principal components (DAPC). Dots represent individuals, and the scatterplot shows only the first two PCs of the DAPC analysis. (**D**) Group memberships of DAPC represented with the cluster colors. (**E**) Contribution of alleles to the first (a) and the second (b) principal components of DAPC. The height of each bar is proportional to the contribution of the corresponding allele to the first and second principal components of the analysis, respectively. Only alleles whose contribution was above a threshold (gray horizontal line) are indicated for the sake of clarity.

Bruvo’s genetic distance among 30 hazelnut genotypes was used to build an Unweighted Pair Group Method with Arithmetic Mean (UPGMA) dendrogram (Figure 2A). The hazelnut germplasm collected in Nebrodi orchards showed two distinctly major clusters. Cluster I grouped 21 genotypes, including six historical local varieties (‘Curcia’, ‘Carrello’, ‘Ghirara’, ‘Minnulara Don Ciccio’, ‘Minnulara Rocco’, and ‘Minnulara’); the landrace ‘Natalina’; twelve LPGB genotypes, of which LPGBCor04 was very close to the variety ‘Ghirara’; and two genotypes identified as ‘Parrinara’ with a different allelic profile at more than one locus. Cluster II grouped nine genotypes, including five more recently released local varieties, ‘Rossa Galvagno’, Panottara Galati Grande’, ‘Enzo’, ‘Panottara Piano Campo’, and ‘Pietro’; two landraces, ‘Tardiva’ and ‘Baratta01’; and two commercial varieties, ‘Tonda Gentile Romana’ and ‘Tonda Gentile delle Langhe’.

To understand the genetic structure of the 30 hazelnut genotypes, a Bayesian clustering analysis was performed and the number of hypothetical groups for each accession was evaluated relating to one or several not predefined groups. To infer the number of groups, the Bayesian process was run with a K value ranging from 1 to 5. The most likely K value was determined using the ΔK method and indicates the maximum change at K = 2 as the most appropriate number of major clusters in Nebrodi hazelnut genotypes (Figure 2B, Table 2). Average distances (expected heterozygosity) between genotypes in the same cluster were 0.8247 for Cluster I and 0.5166 for Cluster II (Table 2).

From the 30 total hazelnut genotypes investigated, 21 individuals (70%) have more than 0.70 membership coefficient (Q) in any given of two genetic clusters. Cluster I grouped two commercial varieties, ‘Tonda Gentile Romana’ and ‘Tonda Gentile delle Langhe’, among the most cultivated commercial varieties in other Italian regions, six Sicilian local varieties, one LPGB genotype (LPGBCor04), and two landraces ‘Baratta01’ and ‘Natalina’. The genotypes included in Cluster II were two older Sicilian local varieties (‘Curcia’ and ‘Parrinara’) still widespread in Nebrodi orchards, eleven LPGB genotypes, and one Sicilian landrace ‘Minnulara Rocco’ (Figure 2B). The local varieties ‘Enzo’ and ‘Minnulara Don Ciccio’ shared similar membership coefficients in two groups, indicating a high degree of admixture.

Additional analysis was performed using discriminant analysis of principal components (DAPC) (Figure 2C). Twenty PCs (80% of variance conserved) of PCA and three discriminant eigenvalues were preserved. Three clusters were identified by the *find.clusters* function. Group 1 comprised a set of fifteen genotypes: eleven LPGB genotypes and ‘Curcia’, ‘Parrinara01’, ‘Parrinara02’, and ‘Natalina’. Group 2 included seven local and two commercial varieties, and one landrace ‘Minnulara Rocco’, while three local varieties and one landrace were drafted in Group 3 (Figure 2C, D). To gain some insight into the underlying causes of the differentiation of 30 hazelnut genotypes, the associated allele loadings were obtained, as shown in Figure 2E. The plot of allele contribution could be useful for a graphical assessment of alleles of major interest and with the largest contribution to this discrimination. The locus CAT-B105 (alleles 156), CATB505 (alleles 122), and CAT-B507 (alleles 182) mostly contributed to the first principal component. Locus CACB020 (allele 284), locus CACB028 (allele 256), locus CATB107 (allele 120), and locus CATB504 (alleles 160 and 178) mostly contributed to the second principal component.

**Figure 3:**
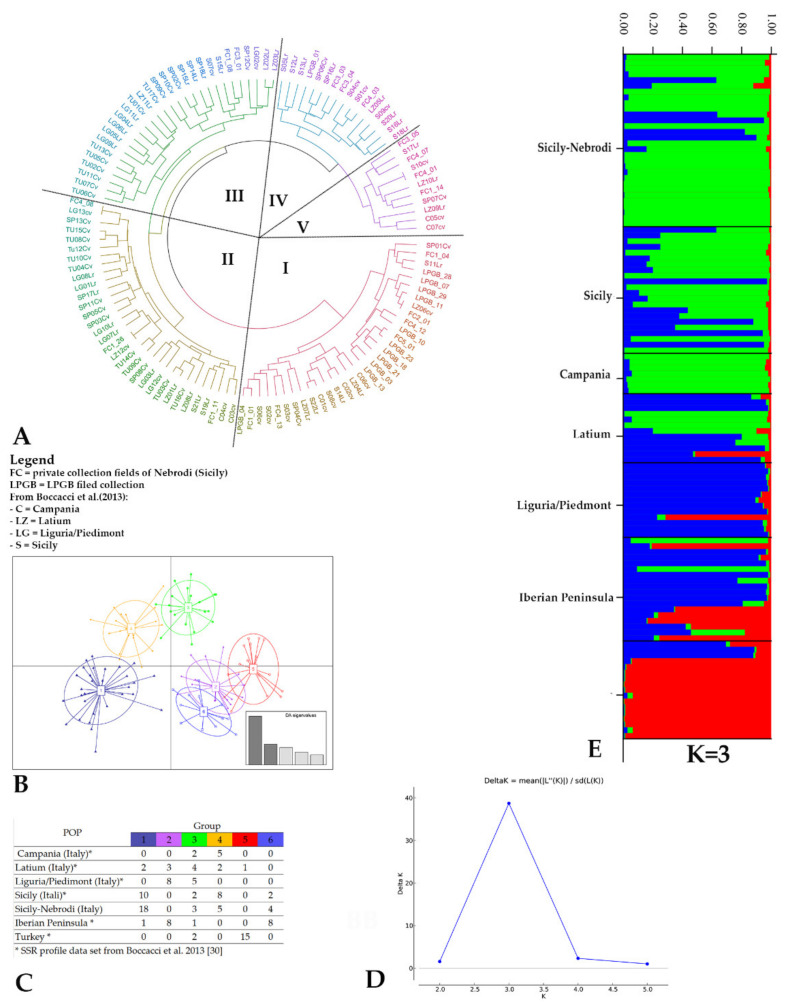
(**A**) UPGMA dendrogram obtained from Italian, Iberian Peninsula, and Turkish genotypes, investigated by Boccacci et al. [28], and the 30 genotypes sampled in Nebrodi Mountains (Sicily, Italy) based on 8 SSR loci and Bruvo’s distance. (**B**) Discriminant analysis of principal components (DAPC) scatter plot. Dots represent individuals, and the scatterplot shows only the first two PCs from the DAPC. (**C**) Group memberships and group sizes obtained from DAPC analysis. (**D**) Delta K according to Evanno et al. 2005. (**E**) STRUCTURE analysis by Bayesian structuring (using STRUCTURE 2.3.4, with a burn-in phase of 10,000 iterations followed by 100,000 MCMC repetitions). Inferred ancestries of 30 genotypes from the Nebrodi area and Italian, Iberian Peninsula, and Turkish germplasm based on 3 genetic groups. Each individual is represented by a vertical colored line. Different colors of each column depict the percent of membership (vertical values on the left of the cluster) of each genotype for four clusters.

To study the relationships among genotypes from the Mediterranean basin, a comparison of our results with already published SSR studies including genotypes from different Italian regions, the Iberian Peninsula, and Turkey (Table S6) was performed based on eight out of nine shared SSR loci and by using UPGMA, DAPC, and STRUCTURE analysis. The UPGMA dendrogram was able to distinguish the Sicilian genotypes from the Nebrodi area from Turkish and Iberian Peninsula genotypes, except for two Spanish genotypes (SP01, SP04) (Figure 3A). 

Cluster I grouped also four local varieties of Nebrodi, ‘Ghirara’ (FC1_01), ‘Curcia’ (FC2_01), ‘Minnulara Don Ciccio’ (FC4_12), ‘Minnulara’ (FC4_13), and the landrace ‘Natalina’ (FC5_01); eleven LPGB genotypes; three genotypes from Latium (LZ04, LZ06, and LZ07); three genotypes from Campania (C01, C02, and C06); and seven genotypes from Sicily (S02, S03, S08, S06, S11, S14, and S22). Only a genotype from the LPBG collection was not included in Cluster I but grouped in Cluster IV. Moreover, the UPGMA analysis showed a synonymy between ‘Curcia’ (FC2_01) and ‘Nocchione’ (LZ06). Cluster II grouped nine genotypes from Turkey and six from the Iberian Peninsula, together with six from Liguria and the commercial varieties ‘Tonda Gentile Romana’ and ‘Tonda Gentile delle Langhe’. Turkish genotypes were subdivided between Clusters II and III. Clusters IV and V included genotypes from Central and Southern Italy.

A similar trend can be observed in the results of the DAPC analysis, where six groups were identified (Figure 3B). Most of the Sicilian genotypes, both from the Nebrodi and those collected in other areas of Sicily, were included in Group 1, clearly distinguished from the Iberian Peninsula and Turkish genotypes. Genotypes from the Iberian Peninsula and Liguria were present in Group 2, while Groups 3 and 4 included genotypes from different geographical areas. 

STRUCTURE analysis resulted in a DeltaK with a peak at K = 3, assigning the genotypes into three clusters (Figure 3D). The genotypes from the Iberian Peninsula mainly shared similar membership coefficients at Cluster III, except for two genotypes, ‘Barcelona’ and ‘Gironell’. The Turkish genotypes were grouped mainly in Cluster II. Sicilian genotypes, as well as those from Campania, were placed mainly in Cluster I (Q > 0.9), except for ‘Pietro’, ‘Tardiva’, ‘Panottara Piano Campo’, and ‘Baratta01’, which grouped in Cluster III together with all the genotypes from Liguria, except for ‘Seigretta’. 

### 2.3. Nut Morphological Analysis

**Figure 4:**
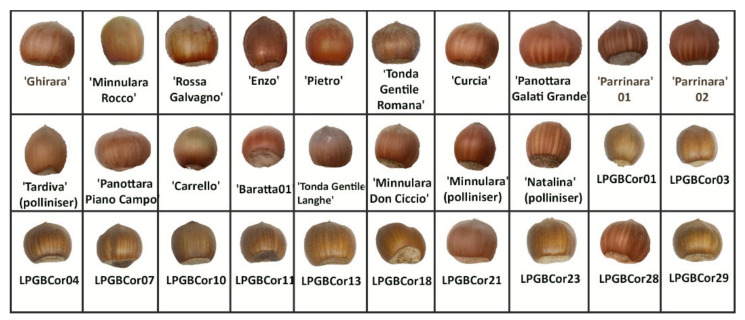
Nut morphology of thirteen Sicilian hazelnut genotypes collected on the Nebrodi Mountains.

For the morphological analysis of nut, we used seven hazelnut descriptors to characterize the 30 hazelnut genotypes of Nebrodi with unique profiles (Figure 4). All collected data from each genotype are reported in Table S7, and the absolute and the relative frequency of each descriptor class are reported in Table S8. The nut size most represented was ‘medium’ (33.33%), followed by ‘small’ (30%). 

Only one local variety (‘Panottara Piano Campo’) produced large nuts classified as ‘very large’. Circular shape (66.6%), light brown color (53.33%), even curvature of basal scar (53.33), and rectangular shape (76.67%) were more frequently observed in the 30 genotypes. The nuts presented more frequently with few or many stripes and an obtuse apex (Figure 4). The genotypes used as pollinizers showed a tendency towards an elongated shape but with different nut colors. Similar morphological characteristics were observed in LPGBCor01 and ‘Minnulara Rocco’ (Figure 4).

**Figure 5:**
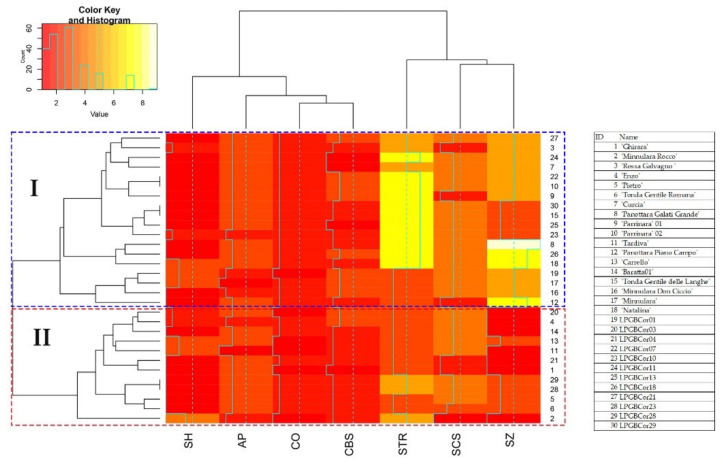
Heatmap clustering of seven nut morphological traits and 30 Sicilian hazelnut genotypes based on Euclidean distances. Red and yellow colors represent reduced and augmented representation levels, respectively. Legend of heatmap is also reported. Cluster I and cluster II are also indicated by dashed rectangles.

Cluster analysis based on Euclidean distance was also displayed as a heatmap (Figure 5), grouping 30 hazelnut genotypes into two main clusters. Each cluster included both local genotype and commercial varieties. The Mantel test was performed to check the correlation between the genetic and morphological distance matrix, highlighting a very low and not significant correlation (r = 0.25; *p* = 0.83).

## 3. Discussion

A better knowledge of crop genetic diversity is crucial to improve yield in sustainable agriculture. Hazelnut germplasm collections were analyzed in different world areas through biochemical [51,52,53], morphological, and genetic characterization using various molecular markers, among which SSRs are the most representative [28,34,38,54]. The development of molecular markers provided increasingly usable tools for DNA-based signature of tree crop varieties [55,56,57]. Here, nine SSR loci, standardized and recommended as replicable DNA-based markers by Biodiversity International [55,58], were employed to investigate the genetic diversity of Sicilian hazelnut germplasm cultivated in the Nebrodi Mountains, making possible the identification of varieties. The genetic relationships between the hazelnut germplasm of the Nebrodi Mountains and the other Italian and European germplasm collections were also investigated.

High-quality DNA is a crucial point for genetic characterization [59]. The most important factor limiting the use of DNA extracted from hazelnut leaf tissue was the rapid time of degradation even if stored at optimal temperatures. Three different DNA extraction procedures, including already-described protocols [60,61] and a commercial kit, were tested to ascertain their effectiveness for extracting high-quality DNA from hazelnut leaves. The modified Martínez-González method [61] has proved to be the best extraction protocol for hazelnut, providing high-quality DNA with a delayed time of degradation, becoming the finest matrix for further molecular analyses.

Cultivated hazelnuts are clonally propagated by farmers, making possible mistakes for attributing each sample to a specific variety. This misclassification could be due to the availability of several individuals from the same or closely related clone, generating redundancy (synonymous and homonymous genotypes) [62].

Our results confirmed many homonymies and synonymies among the most common commercial varieties grown in the Nebrodi Mountains. The high discriminating power of the nine SSR loci was able to clearly distinguish two commercial varieties, thirteen local varieties, and three landraces, as well as twelve unknown genotypes. The ability of the adopted SSR panel was confirmed by the limited improvement of variety identification through the increase in SSR loci [63]. Furthermore, our results agreed with those carried out on international hazelnut collections, including Italian germplasm [28,34,35], albeit with slightly lower discriminating power for some loci. In particular, Boccacci et al. [34] reported a higher discriminating power on average. 

The cultivation of a predominant hazelnut variety named ‘Siciliana’ in Sicily, also known by different local names, such as ‘Nostrale’, ‘Curcia’, ‘Mansa’, or ‘Santa Maria di Gesù’, depending on the area of cultivation, has been already reported [64]. Further studies reported some synonymies between ‘Siciliana’ and other Sicilian varieties, such as ‘Locale di Piazza Armerina’, suggesting that all these varieties may have been clonally propagated from an original variety [34,37]. Our analysis confirmed the synonymy among ‘Santa Maria di Gesù’, ‘Nostrale’ ‘Nocchione’, ‘Comune’, and ‘Siciliana’, all sharing the same SSR profile with ‘Curcia’, a variety little known at the national and international level, but well known and widespread in Sicily. Indeed, the local variety ‘Curcia’ was reported as one of the most widespread in the province of Messina (Sicily) and in other Sicilian areas due to its high productivity [14]. Moreover, the comparison among 30 unique SSR profiles from the Nebrodi Mountains and Sicilian genotypes previously investigated by Boccacci et al. [28] allowed us to identify six distinct profiles: four including local varieties ‘Carrello’, ‘Curcia’, ‘Ghirara’, and ‘Panottara Piano Campo’ (synonymous of ‘Panottara’), and two including the commercial varieties ‘Tonda Gentile Romana’ and ‘Tonda Gentile delle Langhe’. The genetic relationships among accessions presented by hierarchical clustering, structure, and discriminant analysis revealed the distinction between local genotypes and the commercial varieties. UPGMA cluster analysis was able to distinguish two groups; in the first, most local genotypes, still present in old hazelnut orchards, were included together with ‘Curcia’ variety, while more recently selected varieties are grouped in the second cluster. These results can be explained by the widespread presence of the ‘Curcia’ variety in Sicilian orchards since the end of the 1800s [14]. The Bayesian model-based STRUCTURE method and the DAPC furnished similar results, with almost all LPGB genotypes being clustered together with some local varieties, including ‘Curcia’. These observations can be traced back to the long ancient presence in this area of different varieties with similar genetic origin, that generated many synonymies over the decades [14,15]. This is also confirmed by the similarity of almost 50% of LPGB genotypes, collected in the various Nebrodi municipalities, with ‘Curcia’ (SSR profile n.25 in Table S2).

The plant germplasm conservation aims to maintain a high level of genetic diversity, preserving from genetic erosion, both for the presence of high heterozygosity and allelic richness [65]. The Sicilian hazelnut germplasm from the Nebrodi area displayed observed heterozygosity (Ho) values higher than the corresponding expected values at all the loci. These results could be related to the common practice of clonal propagation applied in hazelnut orchards that increase the frequency of multiple alleles at many loci [49]. Future breeding programs could utilize the Sicilian hazelnut germplasm for developing new and more resilient varieties due to the presence of a high level of polymorphism and private allele content. In recent years, the focus of genetic improvement was addressed by using one or a few commercial varieties (‘Tonda Gentile Romana’, ‘Tonda delle Langhe’, and ‘Tonda di Giffoni’), paying limited attention to the narrow genetic diversity in hazelnut. The available studies on agronomic traits of interest among Sicilian hazelnut varieties, such as susceptibility to diseases and fruit qualitative traits, are very limited. Some authors have studied some Sicilian local varieties (‘Agghirara’, ‘Curcia’, ‘Enzo’, ‘Pietro’, and ‘Rossa Galvagno’), here investigated, describing interesting traits such as low susceptibility or resistance to *Phytoptus avellanae*, low sucker emission attitude, nut kernel yield (kernel/nut ratio), round nut shape, and high productivity [66,67]. A characterization of Sicilian genotypes hazelnut germplasm, which should include biochemical traits and nutritional values, is needed before starting breeding. Modern genetic and genomic tools can be used to help the selection of genotypes carrying specific traits. Recently, a high-quality genome assembly has been provided for hazelnut [68,69], which improved the previous draft genome information [70], opening new possibilities for identifying key genes involved in fatty acid biosynthesis, oleic acid accumulation, and biotic stress resistance, which represent important targets for future breeding.

The study of genetic relationships among European hazelnut germplasm collections, according to their geographic origin, revealed that Italian and Iberian Peninsula genotypes clustered together [28,34,38,47]. More recently, the genetic structure of European hazelnut populations has been distinguishing seven main populations: Azerbaijan/Georgia, Central Anatolia, England, Italy, Spain, Black Sea, and Central Europe (Germany, Poland, Moscow) [41]. A closer phylogenetic relationship among all the cultivars from Western and Southern Europe, mostly from Spain and Italy, was then highlighted [71]. A very recent study by Boccacci et al. [72] showed the highest value for K = 3 in the analysis of 181 genotypes from the Iberian and Italian Peninsulas, the British Islands, and the Balkans/Black Sea. The authors reported cultivars from the Iberian Peninsula widespread in all the three groups, thus identifying three gene pools mainly composed of cultivars from Central Europe and the British Islands, Balkans/Black Sea, and the Italian Peninsula, with almost 63% of the genotypes as admixed. Here, we reported an integrated genetic analysis (UPGMA, DAPC, and STRUCTURE) on the Nebrodi area germplasm together with Italian, Iberian, and Turkish genotypes able to determine a redistribution of genetic diversity. STRUCTURE analysis found 77% of Sicilian genotypes clustered together, while this percentage decreased from 54% (DAPC analysis) to 44% detected by UPGMA analysis. Furthermore, the Sicilian genotypes collected in the Nebrodi Mountains were distinguished from the Northern Italian genotypes and even more from Iberian and Turkish genotypes. These results show a more significant distinction of the Sicilian germplasm compared to the previous studies, highlighting a low gene flow between Northern and Southern Italy and more exchange events between the germplasm collections from Southern Italy [28].

The morphological characterization revealed a wide diversity either among the 30 Sicilian genotypes from the Nebrodi Mountains or among hazelnut European genotypes [28,52,73]. The nut traits are highly variable based on the genotype, but also based on agronomic techniques and the environment. The hazelnut cultivars are mainly selected to obtain uniform high-quality nuts for the food industry, focusing on the low incidence of defects, high nut yield (kernel/nut ratio), nut and kernel shape, flavor, and aroma. Nut and kernel shape and size are important traits for confectionery, as sphere-shaped nuts are preferred in the food industry [74]. The circular shape is the most represented in Nebrodi genotypes (67%). Eighty-six percent of these genotypes were of small to medium size of nut and a similar range was reported for many Italian landraces, while the nut size most representative in 46 European cultivars is medium [28]. ‘Tonda Gentile delle Langhe’ and ‘Tonda Gentile Romana’ are recognized as some of the best hazelnut cultivars worldwide, having excellent taste and aroma, reported also for the commercial variety ‘Nocchione’ (synonym of local variety ‘Curcia’) [75]. In addition, morphological traits might be considered for specific food products, chocolate, and pralines, which require smaller and rounder hazelnuts [75]. For these food products, 86% of the Sicilian genotypes from Nebrodi Mountains might be of interest due to their small- and medium-sized nuts. 

Finally, cluster analysis based on genetic distance was not in agreement with that based on Euclidean distance based on morphological nut traits. Indeed, clustering based on Euclidian distance grouped LPGB accessions mainly into one cluster, whereas commercial varieties were distributed in both clusters. 

This result was confirmed by the Mantel test, highlighting that variety identification based only on the morphological traits cannot be considered able to discriminate among hazelnut varieties.

## 4. Materials and Methods

### 4.1. Plant Material and Nut Morphological Traits

**Figure 6:**
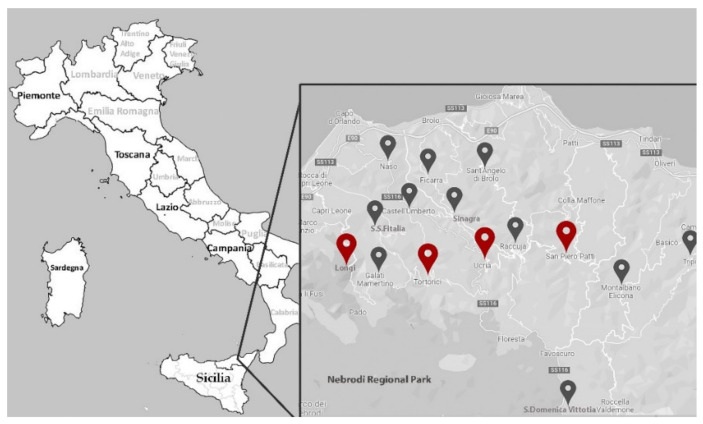
Location sites of hazelnut orchards where LPGB accessions (black pointer) were sampled and of private germplasm collection fields (red pointer).

Seventy-five hazelnut accessions were collected in the Nebrodi Mountains (Sicily, Italy) and analyzed in the present study. This germplasm included 30 LPGB accessions (local varieties/landraces), previously sampled in farms (Figure 6, Table S9), and 45 accessions (local and commercial varieties) sampled in private collection fields (Figure 6, Table S9) and used as reference varieties, some of which are currently registered in the Italian National Register of Plant Variety (Table S9).

Thirty nuts for each genotype were collected for morphological characterization using seven standard UPOV descriptors (Table S10) [76].

### 4.2. DNA Extraction

Young leaf material was sampled from 75 hazelnut accessions. Three extraction protocols were tested to compared DNA extraction yield and purity: (1) modified CTAB-based protocol [60], (2) modified Martínez-González et al. (2017) [61], and (3) NucleoSpin^®^ Plant II commercial kit (Macherey-Nagel, Düren, Germany). Doyle and Doyle’s (1987) method [60] was modified since beta-mercaptoethanol was removed. The protocol reported by Martínez-González et al. (2017) [61], a CTAB-based method, was modified in three steps as follows: (1) incubation in a water bath at 80 °C for 40 min′ of the plant material suspended in the CTAB; (2) elimination of a gelatinous matrix after the precipitation in isopropanol by using a dilution in 200 μL HPLC-grade water to the DNA pellet, immediately eliminated by pipetting; (3) a final incubation of the DNA pellet resuspended in HPLC-grade water at 45 °C for 15 min. A NanoDrop 2000c spectrophotometer was used to quantify and assess the purity of DNA. All extracted DNA was stored at −20 °C. The purity of gDNA was evaluated by PCR amplification of three different SSR markers used in individual and multiplex amplification. The efficiency of PCR amplification was evaluated by checking the height and area of the allele peaks obtained with capillary electrophoresis.

### 4.3. SSR Fingerprinting

Hazelnut genetic characterization was performed using 9 SSR markers (Table S11). The hazelnut DNA amplification was carried out in 3 multiplex PCR sets using the Type-it Microsatellite PCR kit (Qiagen, Hilden, Germany), and the respective forward primers were labeled with the dyes FAM and HEX (Eurofins Genomics, Ebersberg, Germany), as reported in Table S11. Each multiplex PCR reaction was performed in 8 μL total volume including 1.5 μL DNA (50 ng/μL), 2x Qiagen Multiplex PCR master mix buffer, and 0.2 μM primer mix.

The amplifications were carried out using the following PCR cycling conditions: the first regime repeated for 10 cycles involved denaturation at 94 °C for 5 min, followed by denaturation at 94 °C for 3 min, annealing at 60 °C for 1 min and 30 s, and a minute extension at 72 °C, which was lowered by one-degree centigrade for each cycle; the second thermal regime, repeated for 25 cycles, consisted of a denaturation step at 94 °C for 30 s, an annealing cycle at 60 °C, and an extension phase at 72 °C for one minute. Separation and detection of the PCR products were achieved using a 3130 Genetic Analyzer (Applied Biosystems, Foster City, CA, USA) loaded with POP-7 polymer (Applied Biosystem) and the size standard ROX-500 Genescan (Thermo Scientific, Warrington, UK). The determination of the allelic dimensions in terms of base pairs (bp) was carried out using the software GeneMapper Version 4.0.

### 4.4. Statistical Analysis

For duplicate identification, the multi-locus approach was used for genotype matching by program GenAlEx 6.502 [77]. The accessions with different names that were fully matched at the 9 polymorphic SSR loci were considered redundant (duplicates) or synonymously mislabeled accessions. To determine the minimum number of loci necessary to discriminate between individuals, a genotype accumulation curve was calculated by randomly sampling (n = 1000) the nine loci to create the distribution and counting the number of multi-allelic loci by increasing the number of SSRs, using poppr v2.6.0 [78].

The software GenAlEx 6.502 [77] and CERVUS program version 3.0.7 [79] were used to perform genetic analysis of microsatellite profiles, calculating the number of alleles (No), the effective number of alleles per locus (Ne), the observed and expected heterozygosity (Ho, He), the unbiased expected heterozygosity (uHe), the polymorphic information content (PIC), and the number of rare and private alleles [80]. Discriminating power (D) was calculated as reported by Tessir et al. [81] using the poppr R package [78].

Genetic relationships between SSR profiles of genotypes were estimated by using Bruvo’s distance [82] in the poppr R package [78]. A dendrogram was computed from each distance matrix using the UPGMA (Unweighted Pair Group Method with Arithmetic Mean). Investigating genetic diversity using multivariate approaches, a principal components analysis (PCA) was first performed from all accessions, and then a discriminant analysis (DAPC) was performed on the retained principal components [83] to cluster individuals using the R package adegenet [84]. The appropriate number of clusters was inferred using the Bayesian information criterion (BIC), and the number of suitable PCs was identified using the find.clusters function.

Finally, Bayesian-based clustering was performed using STRUCTURE v.2.3.4 [85] to further evaluate the hazelnut germplasm structure. A burn-in period of 10,000 generations and 100,000 Markov chain Monte Carlo replications were used under an admixture model and correlated allele frequencies. Ten replicate simulations were run for each K value, ranging from 1 to 5. 

The most likely K value was processed with STRUCTURE HARVESTER v.0.9.94 [86] and was detected using the Evanno transformation method [87]. To assign samples to clusters, a membership coefficient q > 0.5 was used, while coefficients ≤0.5 were considered genetically admixed. 

For hierarchical clustering based on morphological descriptors collected on nuts, the previously generated Euclidean distance matrix was used, and the average linkage method was applied. A heatmap was generated using the heatmap.2 function from the gplots package of R. The Mantel test [88] was performed to verify possible correlations (through 1000 statistical permutations) between the calculated genetic distances of accessions and the respective nut morphological distances.

## 5. Conclusions

The growing commercial demand for hazelnut derivatives led to the introduction of intensive farming methods that often have a negative impact on the environment. Rural development is still a high priority in many areas worldwide where local communities are mainly dependent on agriculture. Rural communities of Sicilian hazelnut growers require the development of cropping systems based also on the preservation and sustainable use of local agro-biodiversity. Therefore, this study aimed to estimate the genetic diversity of the Sicilian hazelnut germplasm of Nebrodi Mountains and varietal identification by fingerprinting using SSR markers.

The genetic analysis identified 30 unique SSR profiles in the Sicilian germplasm, including both commercial and local varieties. In addition, when compared to Italian and European germplasm previously characterized by the same set of SSRs, the distinctness of Sicilian genotypes was highlighted. Further studies on a broader varietal landscape of cultivated hazelnuts could increase the number of unique alleles, enriching the biodiversity of the hazelnuts available in the Nebrodi area of Sicily.

Our results may help to establish a reference hazelnut germplasm collection from the Nebrodi territory of Sicily and to provide useful tools to produce certified plants. A certified Sicilian hazelnut food chain could provide valuable support to the growth of the hazelnut sector in the future and, at the same time, meet consumer demand for fresh and processed agri-food authentication and traceability. Further studies could contribute to transparency and food safety and allow producers and retailers to properly promote their products.

## Figures and Tables

**Table 1:** Genetic diversity parameters assessed for 9 SSR loci in 30 hazelnut genotypes.

Locus	Na	Ne	Ho	He	uHe	PIC	D
CAC-B020	8	3.051	0.833	0.672	0.684	0.634	0.600
CAC-B028	6	3.523	0.933	0.716	0.728	0.678	0.612
CAT-B105	6	1.532	0.367	0.347	0.353	0.332	0.328
CAT-B107	8	3.488	0.800	0.713	0.725	0.675	0.608
CAT-B501	9	3.147	0.900	0.682	0.694	0.649	0.516
CAT-B502	8	3.383	0.767	0.704	0.716	0.638	0.267
CAT-B504	10	4.174	0.862	0.760	0.774	0.744	0.659
CAT-B505	4	3.186	1.00	0.686	0.698	0.622	0.426
CAT-B507	10	3.922	0.900	0.745	0.758	0.717	0.650
Average	7.67	3.267	0.818	0.670	0.684	0.681	0.518

Na = number of different alleles; Ne = number of effective alleles; Ho = observed heterozygosity; He = expected heterozygosity; uHe = unbiased expected heterozygosity; PIC = polymorphic information content; D = discriminating power.

**Table 2:** The STRUCTURE results of 30 hazelnut genotypes for inferred cluster, the fixation index (Fst), average distances (expected heterozygosity), and number of genotypes assigned to each cluster.

Cluster	Inferred Cluster	Mean Fst	Expected Heterozygosity	Number of Accessions
I	0.460	0.0016	0.8247	13
II	0.540	0.4482	0.5166	17

## Data Availability

Not applicable.

## Supplementary Materials

The following are available online at https://www.mdpi.com/article/10.3390/plants11050631/s1. Figure S1: Genotype accumulation curve in the Sicilian hazelnut germplasm. Proportion of number of multi-locus genotype identified based on the number of loci sampled. There were 1000 randomizations of data analyzed. The band inside the box represents the median (2nd quartile). The dashed red horizontal line denotes the total number of multi-locus genotypes identified in the dataset. Table S1: Comparison of selected DNA extraction methods for hazelnut leaf materials. Table S2: Allelic profiles of 75 hazelnut accessions sampled in field collections of Nebrodi Mountains (Sicily, Italy). Table S3: Thirteen unique profiles obtained by multi-locus match analysis performed on 75 SSR profiles of Sicilian (Nebrodi) hazelnut germplasm. Table S4: Allelic profile of 30 hazelnut genotypes of Nebrodi (Sicily, Italy) obtained after deletion of redundant accessions. Table S5: Rare and private alleles detected in hazelnut germplasm collected in Nebrodi Mountains. Table S6: List of Italian, Iberian Peninsula, and Turkish genotypes investigated by Boccacci et al. (2013) and 30 genotypes sampled in Nebrodi Mountains (Sicily, Italy). Table S7: Qualitative descriptors of nut recorded on 30 genotypes of Sicilian hazelnut germplasm of the Nebrodi Mountains. Table S8: Absolute and relative frequency of qualitative nut traits sampled on 30 Sicilian hazelnut genotypes. Table S9: 75 hazelnut accessions of *Corylus avellana* L. sampled in different field germplasm collections of the Nebrodi Mountains (Sicily, Italy). Table S10: List of descriptors evaluated in 30 nuts for each Sicilian genotypes sampled in the Nebrodi Mountains. Table S11: Summary of the 9 SSR markers, primer multiplex, and co-loading sets used in the study.
