# Supplementary material for: Diversity Assessment and DNA-Based Fingerprinting of Sicilian Hazelnut (Corylus avellana L.) Germplasm
Source: Plants (Basel). 2022 Feb 25;11(5):631. doi: 10.3390/plants11050631 (PMC8912283; doi:10.3390/plants11050631)
Supplement: Supplementary file 1 [file plants-11-00631-s001.zip › plants-1545127-supplementary.pdf]

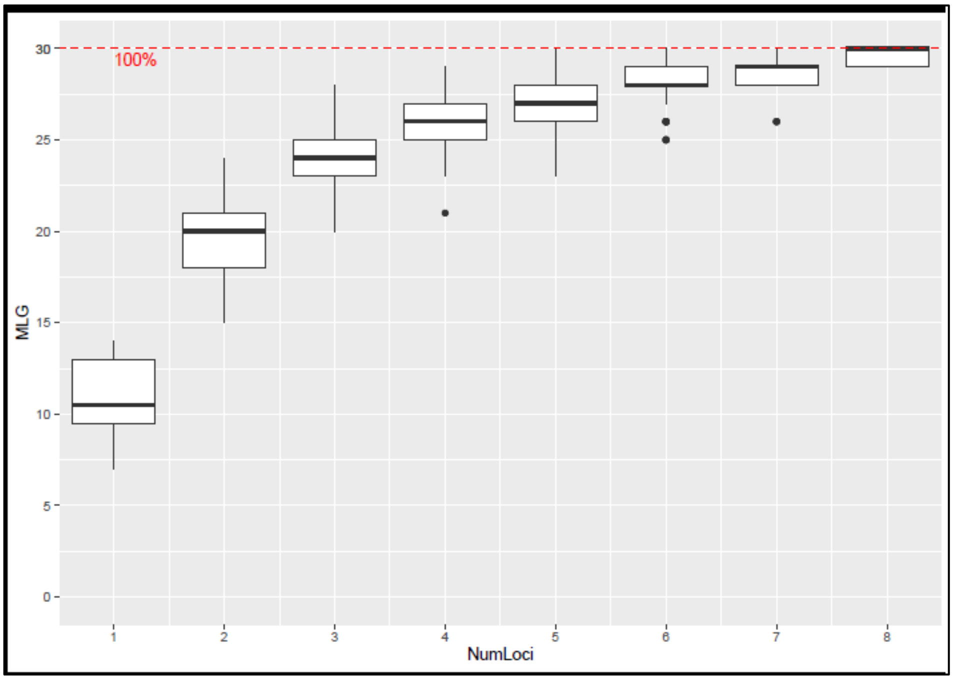

Figure S1. Genotype accumulation curve for Sicilian *Corylus avellana* germplasm. Proportion of multilocus genotypes identified based on the number of loci sampled. There were 1,000 randomizations of the data analyzed. The band inside the box represents the median (2nd quartile). The dashed red horizontal line denotes the total number of multi-locus genotypes identified in the dataset.

Table S1. Comparison of selected DNA extraction methods for hazelnut leaf materials.

Method	Extraction time	Yield	Purity	DNA degradation	Presence of PCR inhibitors
NucleoSpin® Plant II (Macherey-Nagel) kit	3 hrs	low (<50ng/µl)	low	after 1 week	yes
Based CTAB Doyle and Douyle (1987)	8hrs	high (>500ng/µl)	low	after 1 week	yes
Based CTAB Martínez-González et al. (2017)	8hrs	very high (>1 µg/µl)	high	over 12 months	no

Table S2. Allelic profiles of 75 hazelnut accessions sampled in field collections of Nebrodi Mountains (Sicily - Italy).

ID accessions	Cultivar/landrace	CAC-B020		CAC-B028		CAC-B105		CAT-B107		CAT-B501		CAT-B502		CAT-504		CAT-B505		CAT-B507	
FC1_01	'Ghirara'	279	285	257	263	140	156	113	113	116	130	185	187	178	184	120	128	192	196
FC1_02	'Ghirara'	279	285	257	263	140	156	113	113	116	130	185	187	178	184	120	128	192	196
FC1_03	'Ghirara'	279	285	257	263	140	156	113	113	116	130	185	187	178	184	120	128	192	196
FC1_04	'Minnulara Rocco'	279	285	257	263	156	156	113	121	116	130	189	191	160	170	116	128	192	192
FC1_08	'Rossa Galvagno '	285	287	257	263	156	156	113	113	124	130	185	185	170	184	116	128	190	192
FC1_09	'Rossa Galvagno '	285	287	257	263	156	156	113	113	124	130	185	185	170	184	116	128	190	192
FC1_10	'Rossa Galvagno '	285	287	257	263	156	156	113	113	124	130	185	185	170	184	116	128	190	192
FC1_11	'Enzo'	285	287	255	257	156	156	119	123	126	130	191	191	172	184	120	128	190	192
FC1_12	'Enzo'	285	287	255	257	156	156	119	123	126	130	191	191	172	184	120	128	190	192
FC1_13	'Enzo'	285	287	255	257	156	156	119	123	126	130	191	191	172	184	120	128	190	192
FC1_14	'Pietro'	277	277	257	269	156	156	123	123	122	130	185	191	178	178	116	128	180	182
FC1_15	'Pietro'	277	277	257	269	156	156	123	123	122	130	185	191	178	178	116	128	180	182
FC1_16	'Pietro'	277	277	257	269	156	156	123	123	122	130	185	191	178	178	116	128	180	182
FC1_20	'Nostrale Mollese'	279	285	255	263	156	156	113	123	116	130	185	191	160	184	122	128	182	192
FC1_21	'Nostrale Mollese'	279	285	255	263	156	156	113	123	116	130	185	191	160	184	122	128	182	192
FC1_22	'Nostrale Mollese'	279	285	255	263	156	156	113	123	116	130	185	191	160	184	122	128	182	192
FC1_26	'Tonda Gentile Romana'	285	287	263	269	156	162	135	145	124	130	189	193	160	184	120	128	186	190
FC1_27	'Tonda Gentile Romana'	285	287	263	269	156	162	135	145	124	130	189	193	160	184	120	128	186	190
FC1_28	'Tonda Gentile Romana'	285	287	263	269	156	162	135	145	124	130	189	193	160	184	120	128	186	190
FC2_01	'Curcia'	279	285	255	263	156	156	113	123	116	130	185	191	160	184	122	128	182	192
FC2_02	'Curcia'	279	285	255	263	156	156	113	123	116	130	185	191	160	184	122	128	182	192
FC2_03	'Curcia'	279	285	255	263	156	156	113	123	116	130	185	191	160	184	122	128	182	192
FC3_01	'Panottara Galati Grande'	285	287	263	279	148	156	113	121	124	130	185	185	170	184	116	128	180	192
FC3_02	'Panottara Galati Grande'	285	287	263	279	148	156	113	121	124	130	185	185	170	184	116	128	180	192
FC3_03	'Parrinara'	285	285	255	269	156	156	119	123	116	130	191	191	160	178	120	128	182	182
FC3_04	'Parrinara'	285	285	255	269	156	156	119	123	116	130	191	199	160	184	120	122	180	192
FC3_05	'Tardiva' (polliniser)	287	287	257	257	156	156	121	123	118	124	185	191	178	178	116	120	182	188
FC3_06	'Tardiva' (polliniser)	287	287	257	257	156	156	121	123	118	124	185	191	178	178	116	120	182	188
FC4_01	'Panottara Piano Campo'	275	279	263	267	140	156	121	121	116	122	187	203	174	180	122	128	180	182
FC4_02	'Panottara Piano Campo'	275	279	263	267	140	156	121	121	116	122	187	203	174	180	122	128	180	182
FC4_03	'Carrello'	279	287	257	263	140	156	123	145	116	116	185	191	160	170	116	128	180	192
FC4_04	'Carrello'	279	287	257	263	140	156	123	145	116	116	185	191	160	170	116	128	180	192
FC4_05	'Carrello'	279	287	257	263	140	156	123	145	116	116	185	191	160	170	116	128	180	192
FC4_06	'Ghirara'	279	285	255	263	156	156	113	123	116	130	185	191	160	184	122	128	182	192
FC4_07	'Ghirara'	279	285	257	257	156	156	121	129	124	134	177	185	170	184	116	122	182	192
FC4_08	'Santa Maria di Gesù'	283	285	257	263	156	156	135	153	130	130	187	191	172	184	116	128	186	192
FC4_09	'Santa Maria di Gesù'	279	285	255	263	156	156	113	123	116	130	185	191	160	184	122	128	182	192
FC4_10	'Minnulara Don Ciccio'	279	285	255	263	156	156	113	123	116	132	185	191	160	184	122	128	182	192

FC4_11	'Minnulara Don Ciccio'	279	285	255	263	156	156	113	123	116	132	185	191	160	184	122	128	182	192
FC4_12	'Minnulara Don Ciccio'	285	287	257	263	140	156	113	123	116	130	185	185	160	176	120	128	182	192
FC4_13	'Minnulara' (polliniser)	281	289	255	257	140	150	113	123	116	130	185	191	-1	-1	122	128	190	196
FC5_01	'Natalina' (polliniser)	279	285	255	263	156	156	113	123	114	116	185	191	160	170	122	128	182	192
FC5_02	'Curcia'	279	285	255	263	156	156	113	123	116	130	185	191	160	184	122	128	182	192
FC5_03	'Curcia'	279	285	255	263	156	156	113	123	116	130	185	191	160	184	122	128	182	192
FC5_04	'Curcia'	279	285	255	263	156	156	113	123	116	130	185	191	160	184	122	128	182	192
LPGBCor01	Cor01	279	285	255	263	150	156	123	123	112	130	191	193	160	160	122	128	182	182
LPGBCor02	Cor02	279	285	255	263	150	156	123	123	112	130	191	193	160	160	122	128	182	182
LPGBCor03	Cor03	279	285	255	263	156	156	113	123	116	130	185	191	160	184	122	128	174	178
LPGBCor04	Cor04	279	285	255	263	140	156	113	113	116	130	187	187	178	184	120	128	192	196
LPGBCor05	Cor05	279	285	255	263	156	156	113	123	116	130	185	191	160	184	122	128	182	192
LPGBCor06	Cor06	279	285	255	263	156	156	113	123	116	130	185	191	160	184	122	128	182	192
LPGBCor07	Cor07	279	285	255	263	156	156	113	123	116	130	189	189	160	184	122	128	182	192
LPGBCor08	Cor08	279	285	255	263	156	156	113	123	116	130	185	191	160	184	122	128	182	192
LPGBCor09	Cor09	279	285	255	263	156	156	113	123	116	130	185	191	160	184	122	128	182	192
LPGBCor10	Cor10	285	285	255	257	156	156	113	123	116	130	185	191	160	178	122	128	182	192
LPGBCor11	Cor11	279	285	255	263	154	156	113	123	116	130	185	191	160	184	122	128	180	192
LPGBCor12	Cor12	279	285	255	263	156	156	113	123	116	130	185	191	160	184	122	128	182	192
LPGBCor13	Cor13	279	285	255	263	156	156	113	123	116	122	185	191	160	184	122	128	182	192
LPGBCor14	Cor14	279	285	255	263	156	156	113	123	116	130	185	191	160	184	122	128	182	192
LPGBCor15	Cor15	279	285	255	263	156	156	113	123	116	130	185	191	160	184	122	128	182	192
LPGBCor16	Cor16	279	285	255	263	156	156	113	123	116	130	185	191	160	184	122	128	182	192
LPGBCor17	Cor17	279	285	255	263	156	156	113	123	116	130	189	189	160	184	122	128	182	192
LPGBCor18	Cor18	279	285	255	263	156	156	113	123	116	130	185	191	160	162	122	128	182	192
LPGBCor19	Cor19	279	285	255	263	156	156	113	123	116	130	185	191	160	162	122	128	182	192
LPGBCor20	Cor20	279	285	255	263	156	156	113	123	116	130	185	191	160	184	122	128	182	192
LPGBCor21	Cor21	279	285	255	263	156	156	113	123	116	130	185	191	160	160	122	128	182	192
LPGBCor22	Cor22	279	285	255	263	156	156	113	123	116	130	185	191	160	184	122	128	182	192
LPGBCor23	Cor23	279	285	255	263	156	156	113	123	116	116	185	191	160	164	122	128	182	192
LPGBCor24	Cor24	279	285	255	263	156	156	113	123	116	130	185	191	160	184	122	128	182	192
LPGBCor25	Cor25	279	285	255	263	156	156	113	123	116	130	185	191	160	184	122	128	182	192
LPGBCor26	Cor26	279	285	255	263	156	156	113	123	116	130	185	191	160	184	122	128	182	192
LPGBCor27	Cor27	279	285	255	263	156	156	113	123	116	130	185	191	160	184	122	128	182	192
LPGBCor28	Cor28	279	285	255	263	156	156	119	123	116	130	191	199	160	184	122	128	182	192
LPGBCor29	Cor29	279	285	255	263	154	156	113	123	116	130	185	191	160	184	116	128	182	192
LPGBCor30	Cor30	279	285	255	263	154	156	113	123	116	130	185	191	160	184	116	128	182	192

Table S3. Thirteen unique profiles obtained by multilocus match analysis performed on 75 SSR profiles of Sicilian (Nebrodi) hazelnut germplasm.

Profile	ID germplasm Bank	Accession name	accession type	Nut Shape
1	FC4_13	'Minnulara' (polliniser)	local variety	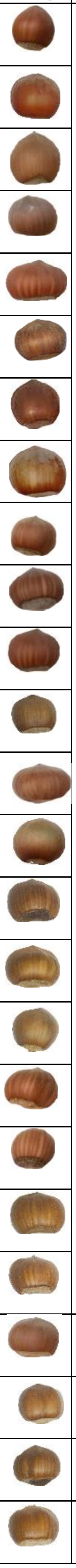
2	FC1_14, FC1_15, FC1_16	'Pietro'	local variety	
3	FC3_05, FC3_06	'Tardiva' (polliniser)	local variety	
4	FC4_08	'Tonda Gentile delle Langhe'	commercial variety	
5	FC3_01, FC3_02	'Panottara Galati Grande'	local variety	
6	FC1_26, FC1_27, FC1_28	'Tonda Gentile Romana'	commercial variety	
7	FC1_11, FC1_12, FC1_13	'Enzo'	local variety	
8	FC1_08, FC1_09, FC1_10	'Rossa Galvagno'	local variety	
9	FC4_12	'Minnulara Don Ciccio'	local variety	
10	FC3_04	'Parrinara'01	local variety	
11	FC3_03	'Parrinara'02	local variety	
12	LPGBCor10	Cor10	unknown landrace	
13	FC4_01, FC4_02	'Panottara Piano Campo'	local variety	
14	FC4_03, FC4_04, FC4_05	'Carrello'	local variety	
15	LPGBCor11	Cor11	unknown landrace	
16	LPGBCor29, LPGBCor30	Cor29	unknown landrace	
17	LPGBCor01, LPGBCor02	Cor01	unknown landrace	
18	LPGBCor28	Cor28	unknown landrace	
19	FC5_01	'Natalina' (polliniser)	landrace	
20	LPGBCor13	Cor13	unknown landrace	
21	LPGBCor18. LPGBCor19	Cor18	unknown landrace	
22	LPGBCor21	Cor21	unknown landrace	
23	LPGBCor03	Cor03	unknown landrace	
24	LPGBCor07	Cor07	unknown landrace	
25	LPGBCor23	Cor23	unknown landrace	
26	FC1_20, FC1_21, FC1_22	'Nostrale Mollese'	local variety	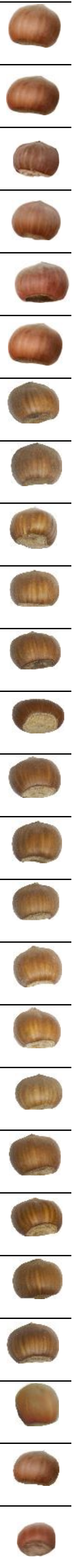
	FC2_01, FC2_02, FC2_03	'Curcia'	local variety	
	FC4_06	'Ghirara'	local variety	
	FC4_09	'Santa Maria di Gesù'	local variety	
	FC4_10, FC4_11	'Minnulara Don Ciccio' *	local variety	
	FC5_02, FC5_03, FC5_04	'Curcia'	local variety	
	LPGBCor05	Cor05	unknown landrace	
	LPGBCor06	Cor06	unknown landrace	
	LPGBCor08	Cor08	unknown landrace	
	LPGBCor09	Cor09	unknown landrace	
	LPGBCor12	Cor12	unknown landrace	
	LPGBCor14	Cor14	unknown landrace	
	LPGBCor15	Cor15	unknown landrace	
	LPGBCor16	Cor16	unknown landrace	
	LPGBCor17	Cor17*	unknown landrace	
	LPGBCor20	Cor20	unknown landrace	
	LPGBCor22	Cor22	unknown landrace	
	LPGBCor24	Cor24	unknown landrace	
	LPGBCor25	Cor25	unknown landrace	
	LPGBCor26	Cor26	unknown landrace	
	LPGBCor27	Cor27	unknown landrace	
27	LPGBCor04	Cor04	unknown landrace	
28	FC1_04	Minnulara Rocco'	landrace	
29	FC1_01, FC1_02, FC1_03	Ghirara'	local variety	
30	FC4_07	'Baratta01'	landrace	

* 2 bp different in one allele

Table S4. Allelic profile of 30 hazelnut genotypes of Nebrodi (Sicily-Italy) obtained after deletion of redundant accessions.

ID germplasm Bank	Accession name	CAC-B020		CAC-B028		CAC-B105		CAT-B107		CAT-B501		CAT-B502		CAT-B504		CAT-B505		CAT-B507	
FC1_01	'Ghirara'	279	285	257	263	140	156	113	113	116	130	185	187	178	184	120	128	192	196
FC1_04	'Minnulara Rocco'	279	285	257	263	156	156	113	121	116	130	189	191	160	170	116	128	192	192
FC1_08	'Rossa Galvagno '	285	287	257	263	156	156	113	113	124	130	185	185	170	184	116	128	190	192
FC1_11	'Enzo'	285	287	255	257	156	156	119	123	126	130	191	191	172	184	120	128	190	192
FC1_14	'Pietro'	277	277	257	269	156	156	123	123	122	130	185	191	178	178	116	128	180	182
FC1_26	'Tonda Gentile Romana'	285	287	263	269	156	162	135	145	124	130	189	193	160	184	120	128	186	190
FC2_01	'Curcia'	279	285	255	263	156	156	113	123	116	130	185	191	160	184	122	128	182	192
FC3_01	'Panottara Galati Grande'	285	287	263	279	148	156	113	121	124	130	185	185	170	184	116	128	180	192
FC3_03	'Parrinara' 01*	285	285	255	269	156	156	119	123	116	130	191	191	160	178	120	128	182	182
FC3_04	'Parrinara' 02*	285	285	255	269	156	156	119	123	116	130	191	199	160	184	120	122	180	192
FC3_05	'Tardiva'	287	287	257	257	156	156	121	123	118	124	185	191	178	178	116	120	182	188
FC4_01	'Panottara Piano Campo'	275	279	263	267	140	156	121	121	116	122	187	203	174	180	122	128	180	182
FC4_03	'Carrello'	279	287	257	263	140	156	123	145	116	116	185	191	160	170	116	128	180	192
FC4_07	Baratta01**	279	285	257	257	156	156	121	129	124	134	177	185	170	184	116	122	182	192
FC4_08	'Tonda Gentile delle Langhe'	283	285	257	263	156	156	135	153	130	130	187	191	172	184	116	128	186	192
FC4_12	'Minnulara Don Ciccio'	285	287	257	263	140	156	113	123	116	130	185	185	160	176	120	128	182	192
FC4_13	'Minnulara'	281	289	255	257	140	150	113	123	116	130	185	191	-1	-1	122	128	190	196
FC5_01	'Natalina'	279	285	255	263	156	156	113	123	114	116	185	191	160	170	122	128	182	192
LPGB_01	LPGBCor01	279	285	255	263	150	156	123	123	112	130	191	193	160	160	122	128	182	182
LPGB_03	LPGBCor03	279	285	255	263	156	156	113	123	116	130	185	191	160	184	122	128	174	178
LPGB_04	LPGBCor04	279	285	255	263	140	156	113	113	116	130	187	187	178	184	120	128	192	196
LPGB_07	LPGBCor07	279	285	255	263	156	156	113	123	116	130	189	189	160	184	122	128	182	192
LPGB_10	LPGBCor10	285	285	255	257	156	156	113	123	116	130	185	191	160	178	122	128	182	192
LPGB_11	LPGBCor11	279	285	255	263	154	156	113	123	116	130	185	191	160	184	122	128	180	192
LPGB_13	LPGBCor13	279	285	255	263	156	156	113	123	116	122	185	191	160	184	122	128	182	192
LPGB_18	LPGBCor18	279	285	255	263	156	156	113	123	116	130	185	191	160	162	122	128	182	192
LPGB_21	LPGBCor21	279	285	255	263	156	156	113	123	116	130	185	191	160	160	122	128	182	192
LPGB_23	LPGBCor23	279	285	255	263	156	156	113	123	116	116	185	191	160	164	122	128	182	192
LPGB_28	LPGBCor28	279	285	255	263	156	156	119	123	116	130	191	199	160	184	122	128	182	192
LPGB_29	LPGBCor29	279	285	255	263	154	156	113	123	116	130	185	191	160	184	116	128	182	192

* No published allelic profile is available for commercial variety ‘Parrinara'

**accession with allelic profile not matching the reference varieties

Table S5. Rare and private alleles detected in hazelnut germplasm collected in Nebrodi Mountains.

Locus	Allele	Hets	Homs	Freq
CAC-B020	275	'Panottara Piano Campo'		0.0156
	277		'Pietro'	0.0313
	281	'Minnulara' (polliniser)		0.0156
	283	'Tonda Gentile delle Langhe'		0.0156
	289	'Minnulara' (polliniser)		0.0156
CAC- B028	267	'Panottara Piano Campo'		0.0156
	279	'Panottara Grande'		0.0313
CAC-B105	148	'Panottara Grande'		0.0156
	150	'Minnulara' (polliniser) - LPGBCor02		0.0313
	154	LPGBCor11 - LPGBCor29		0.0313
	162	'Tonda Gentile Romana'		0.0156
CAT-B107	129	Baratta 01		0.0156
	135	'Tonda Gentile delle Langhe'- 'Tonda Gentile Romana'		0.0313
	145	'Carrello' - 'Tonda Gentile Romana'		0.0313
	153	'Tonda Gentile delle Langhe'		0.0156
CAT-B501	112	LPGBCor01		0.0156
	114	'Natalina'		0.0156
	118	'Tardiva' (polliniser)		0.0156
	122	'Pietro' - LPGBCor13 - 'Panottara Piano Campo'		0.0469
	126	'Enzo'		0.0156
	134	Baratta 01		0.0156
CAT-B502	177	Baratta 01		0.0156
	193	Baratta 01 - 'Tonda Gentile Romana'		0.0313
	199	'Parrinara'02 - LPBGCor28		0.0313
	203	'Panottara Piano Campo'		0.0156
CAT-B504*	162	LPGBCor18		0.0323
	164	LPGBCor23		0.0161
	172	'Tonda Gentile delle Langhe' - 'Enzo'		0.0323
	174	'Panottara Piano Campo'		0.0161
	176	'Minnulara Don Ciccio '		0.0161
	180	'Panottara Piano Campo'		0.0161
CAT-B507	174	LPGBCor03		0.0156
	178	LPGBCor03		0.0156
	188	'Tardiva'(polliniser)		0.0156

Hets =heterozygous state

Homs = homozygous state

* locus with one missing data

Table S6. List of Italian, Iberian Peninsula and Turkish genotypes investigated by Boccacci et al (2013) and 30 genotypes sampled in Nebrodi Mountains (Sicily-Italy).

ID	Accession name	Type	Origin	ID	Accession name	Type	Origin
C01	Camponica	CV	Campania	FC2_01	Curcia	CV	Sicily (Nebrodi Park)
C02	Mortarella	CV	Campania	FC3_01	Panottara Galati Grande	CV	Sicily (Nebrodi Park)
C03	Riccia di Talanico	CV	Campania	FC3_03	Parrinara 01	CV	Sicily (Nebrodi Park)
C04	San Giovanni	CV	Campania	FC3_04	Parrinara02	CV	Sicily (Nebrodi Park)
C05	Tonda bianca	CV	Campania	FC3_05	Tardiva	LR	Sicily (Nebrodi Park)
C06	Tonda di Giffoni	CV	Campania	FC4_01	Panottara Piano Campo	CV	Sicily (Nebrodi Park)
C07	Tonda rossa	CV	Campania	FC4_03	Carrello	CV	Sicily (Nebrodi Park)
LZ01	Allungata	LR	Latium	FC4_07	Baratta01	LR	Sicily (Nebrodi Park)
LZ02	Barrettona	LR	Latium	FC4_08	Tonda Gentile delle Langhe	CV	Sicily (Nebrodi Park)
LZ03	Cappello del prete	LR	Latium	FC4_12	Minnulara Don Ciccio	CV	Sicily (Nebrodi Park)
LZ04	Itavex	LR	Latium	FC4_13	Minnulara	LR	Sicily (Nebrodi Park)
LZ05	Madonnella	LR	Latium	FC5_01	Natalina	LR	Sicily (Nebrodi Park)
LZ06	Nocchione	CV	Latium	LPGBCor01	Cor01	LR	Sicily (Nebrodi Park)
LZ07	Nocciola Ada	LR	Latium	LPGBCor03	Cor03	LR	Sicily (Nebrodi Park)
LZ08	Nocciola Benedetta	LR	Latium	LPGBCor04	Cor04	LR	Sicily (Nebrodi Park)
LZ09	Nocciola centenaria	LR	Latium	LPGBCor07	Cor07	LR	Sicily (Nebrodi Park)
LZ10	Nocciola lunga	LR	Latium	LPGBCor10	Cor10	LR	Sicily (Nebrodi Park)
LZ11	San Vicino Vittori	LR	Latium	LPGBCor11	Cor11	LR	Sicily (Nebrodi Park)
LZ12	Tonda Gentile Romana	CV	Latium	LPGBCor13	Cor13	LR	Sicily (Nebrodi Park)
LG01	Bardina	LR	Liguria	LPGBCor18	Cor18	LR	Sicily (Nebrodi Park)
LG02	Catainetto	CV	Liguria	LPGBCor21	Cor21	LR	Sicily (Nebrodi Park)
LG03	Ciasetta	LR	Liguria	LPGBCor23	Cor23	LR	Sicily (Nebrodi Park)
LG04	Del Rosso	LR	Liguria	LPGBCor28	Cor28	LR	Sicily (Nebrodi Park)
LG05	Dell'Orto	LR	Liguria	LPGBCor29	Cor29	LR	Sicily (Nebrodi Park)
LG06	Gianchetta	LR	Liguria	SP01CL	Barcelona	CV	Spain
LG07	Lunghera	LR	Liguria	SP02CL	Casina	CV	Spain
LG08	Menoia	LR	Liguria	SP03CL	Comum	CV	Portugal
LG09	Noscello	LR	Liguria	SP04CL	Culplà	CV	Spain
LG10	Seigretta	LR	Liguria	SP05CL	Da Viega	CV	Portugal
LG11	Tapparona	LR	Liguria	SP06CL	Gironell	CV	Spain
LG12	Trietta	CV	Liguria	SP07CL	Grifoll	CV	Spain
LG13	Tonda Gentile delle Langhe	CV	Piedmont	SP08CL	Morell	CV	Spain
S01	Carrello	CV	Sicily*	SP09CL	Negret	CV	Spain
S02	Ghirara	CV	Sicily*	SP10CL	Pauetet	CV	Spain
S03	Iannusa Racinante	CV	Sicily*	SP11CL	Ribet	CV	Spain
S04	Minnulara	CV	Sicily*	SP12CL	Segorbe	CV	Spain
S05	Minnulara	LR	Sicily*	SP13CL	Trenet	CV	Spain
S06	Minnulara Don Ciccio	CV	Sicily*	SP14LR	Allande	LR	Spain
S07	Napoletana	CV	Sicily*	SP15LR	Las Cuevas	LR	Spain
S08	Napoletanedda	CV	Sicily*	SP16LR	Priero	LR	Spain
S09	Nociara	CV	Sicily*	SP17LR	Quinta Rego	LR	Portugal
S10	Panottara	CV	Sicily*	SP18LR	Robriguedo	LR	Spain
S11	Selvaggiola agostara	LR	Sicily (Etna Park)	TU01	Acı	CV	Turkey
S12	Selvaggiola lunga	LR	Sicily (Etna Park)	TU02	Allahverdi	CV	Turkey
S13	Selvaggiola riccia	LR	Sicily (Etna Park)	TU03	Badem	CV	Turkey
S14	Selvaggiola SIC13	LR	Sicily (Etna Park)	TU04	Çakıldak	CV	Turkey
S15	Selvaggiola SIC16	LR	Sicily (Etna Park)	TU05	Foşa	CV	Turkey
S16	Selvaggiola SIC17	LR	Sicily (Etna Park)	TU06	Incekara	CV	Turkey
S17	Selvaggiola SIC4	LR	Sicily (Etna Park)	TU07	Kalınkara	CV	Turkey
S18	Selvaggiola SIC6	LR	Sicily (Etna Park)	TU08	Kan	CV	Turkey
S19	Selvaggiola SIC7	LR	Sicily (Etna Park)	TU09	Karafındık	CV	Turkey
S20	Selvaggiola tardiva SIC12	LR	Sicily (Etna Park)	TU10	Kargalak	CV	Turkey
S21	Selvaggiola tardiva SIC8	LR	Sicily (Etna Park)	TU11	Kuş	CV	Turkey
S22	Trichette	LR	Sicily (Etna Park)	TU12	Palaz	CV	Turkey
FC1_01	Ghirara	CV	Sicily (Nebrodi Park)	TU13	Sivri	CV	Turkey
FC1_04	Minnulara Rocco	LR	Sicily (Nebrodi Park)	TU14	Tombul	CV	Turkey
FC1_08	Rossa Galvagno	CV	Sicily (Nebrodi Park)	TU15	Uzunmusa	CV	Turkey
FC1_11	Enzo	CV	Sicily (Nebrodi Park)	TU16	Yassı Badem	CV	Turkey
FC1_14	Pietro	CV	Sicily (Nebrodi Park)	TU17	Yuvarlak Badem	CV	Turkey
FC1_26	Tonda Gentile Romana	CV	Sicily (Nebrodi Park)				

* cultivars present in different Italian field collections

Table S7. Qualitative descriptors of nut recorded on 30 genotypes of Sicilian hazelnut germplasm of Nebrodi Mountains.

ID	Variety/landrace	Size	Shape	Shell colour	Curvature of nut basal scar	Shape of cross section	Shell striping	Shape of nut apex
FC1_01	'Ghirara'	1	1	1	1	2	3	3
FC1_04	'Minnulara Rocco'	1	4	1	2	1	5	2
FC1_08	'Rossa Galvagno '	5	2	2	2	2	5	3
FC1_11	'Enzo'	1	2	2	3	4	3	2
FC1_14	'Pietro'	3	1	2	2	4	3	3
FC1_26	'Tonda Gentile Romana'	3	1	2	2	3	3	3
FC2_01	'Curcia'	5	1	2	1	4	5	3
FC3_01	'Panottara Galati Grande'	9	1	2	2	4	7	3
FC3_03	'Parrinara'01	5	1	2	3	2	7	3
FC3_04	'Parrinara'02	5	1	2	3	4	7	3
FC3_05	'Tardiva'	1	3	1	2	4	3	1
FC4_01	'Panottara Piano Campo'	7	1	2	2	2	3	3
FC4_03	'Carrello'	3	3	1	2	4	3	3
FC4_07	Baratta01	1	1	2	2	4	3	3
FC4_08	'Tonda Gentile delle Langhe'	3	1	2	2	4	7	3
FC4_12	'Minnulara Don Ciccio'	5	1	2	3	4	3	2
FC4_13	'Minnulara'	5	3	2	3	4	3	1
FC5_01	'Natalina'	7	3	2	2	4	7	3
LPGB_01	LPGBCor01	5	3	1	3	4	3	2
LPGB_03	LPGBCor03	1	2	1	3	4	3	3
LPGB_04	LPGBCor04	1	1	2	2	2	3	3
LPGB_07	LPGBCor07	5	1	2	3	4	7	3
LPGB_10	LPGBCor10	3	2	2	2	4	7	2
LPGB_11	LPGBCor11	5	1	3	1	4	7	3
LPGB_13	LPGBCor13	3	1	4	1	4	7	3
LPGB_18	LPGBCor18	7	1	5	3	4	7	3
LPGB_21	LPGBCor21	5	1	6	3	4	5	3
LPGB_23	LPGBCor23	3	1	7	2	4	5	3
LPGB_28	LPGBCor28	3	1	8	2	4	5	3
LPGB_29	LPGBCor29	3	1	9	2	4	7	3

Table S8. Absolute and relative frequency of qualitative nut traits sampled on 30 Sicilian hazelnut genotypes.

Descriptor	Descriptor state	Absolute frequency	Percent
**size**	very small	7	23.33
	small	9	30.00
	medium	10	33.33
	large	3	10.00
	very large	1	3.33
**shape**	circular	20	66.67
	triangular	4	13.33
	ovate	5	16.67
	oblong	1	3.33
**shell colour**	greenish yellow	4	13.33
	light brown	16	53.33
	brown	10	33.33
**curvature of basal scar**	concave	4	13.33
	even	16	53.33
	convex	10	33.33
**shape of cross section**	elliptic	1	3.33
	circular	5	16.67
	triangular	1	3.33
	rectangular	23	76.67
**shell striping**	few	13	43.33
	medium	6	20.00
	many	11	36.67
**shape of apex**	narrow aute	2	6.67
	broad acute	5	16.67
	obtuse	23	76.67

Table S9. 75 hazelnut accessions of *Corylus avellana* L. sampled in diffrent field germplasm collections of Nebrodi Mountains (Sicily - Italy).

ID accession*	Field Collection name	variety/landrace	Municipality	Accession tpye	Registred **
FC1_01	GALVAGNO	'Ghirara'	San Piero Patti	Commercial variety **	yes
FC1_02		'Ghirara'	San Piero Patti		yes
FC1_03		'Ghirara'	San Piero Patti		yes
FC1_04		'Minnulara Rocco'	San Piero Patti	Local variety**	no
FC1_08		'Rossa Galvagno '	San Piero Patti	Local variety**	yes
FC1_09		'Rossa Galvagno '	San Piero Patti		yes
FC1_10		'Rossa Galvagno '	San Piero Patti		yes
FC1_11		'Enzo'	San Piero Patti	Local variety**	yes
FC1_12		'Enzo'	San Piero Patti		yes
FC1_13		'Enzo'	San Piero Patti		yes
FC1_14		'Pietro'	San Piero Patti	Local variety**	yes
FC1_15		'Pietro'	San Piero Patti		yes
FC1_16		'Pietro'	San Piero Patti		yes
FC1_17		'Nostrale Mollese'	San Piero Patti	Commercial variety**	yes
FC1_18		'Nostrale Mollese'	San Piero Patti		yes
FC1_19		'Nostrale Mollese'	San Piero Patti		yes
FC1_20		'Tonda Gentile Romana'	San Piero Patti	Commercial variety**	yes
FC1_21		'Tonda Gentile Romana'	San Piero Patti		yes
FC1_22		'Tonda Gentile Romana'	San Piero Patti		yes
FC2_01	FLORENA	'Curcia'	Ucria	Local variety	yes
FC2_02		'Curcia'	Ucria		yes
FC2_03		'Curcia'	Ucria		yes
FC3_01	GALATI	'Panottara Galati Grande'	Tortorici	Local variety**	yes
FC3_02		'Panottara Galati Grande'	Tortorici		yes
FC3_03		'Parrinara'	Tortorici	Local variety**	yes
FC3_04		'Parrinara'	Tortorici		yes
FC3_05		'Tardiva' (polliniser)	Tortorici	Local variety**	no
FC3_06		'Tardiva' (polliniser)	Tortorici		no
FC4_01	BARATTA	'Panottara' (Piano Campo)	Ucria	Local variety**	yes
FC4_02		'Panottara' (Piano Campo)	Ucria		yes
FC4_03		'Carrello'	Ucria	Local variety**	yes
FC4_04		'Carrello'	Ucria		yes
FC4_05		'Carrello'	Ucria		yes
FC4_06		'Ghirara'	Ucria	Local variety**	yes
FC4_07		'Ghirara'	Ucria		yes
FC4_08		'Santa Maria di Gesù'	Ucria	Local variety**	yes
FC4_09		'Santa Maria di Gesù'	Ucria		yes
FC4_10		'Minnulara Don Ciccio'	Ucria	Local variety**	yes
FC4_11		'Minnulara Don Ciccio'	Ucria		yes
FC4_12		'Minnulara Don Ciccio'	Ucria		yes
FC4_13		'Minnulara' (polliniser)	Ucria	Local variety**	no
FC5_01	LONGI	'Natalina' (polliniser)	Longi	Landrcace	no
FC5_02		'Curcia'	Longi	Local variety	yes
FC5_03		'Curcia'	Longi		yes
FC5_04		'Curcia'	Longi		yes
LPGB_01	LPGB	Cor_01	Ucria	indeterminate	
LPGB_02		Cor_02	Ucria		
LPGB_03		Cor_03	Ucria		
LPGB_04		Cor_04	Ucria		
LPGB_05		Cor_05	Ucria		
LPGB_06		Cor_06	Ucria		
LPGB_07		Cor_07	Ucria		
LPGB_08		Cor_08	Ucria		
LPGB_09		Cor_09	Ucria		
LPGB_10		Cor_10	Ucria		
LPGB_11		Cor_11	Ucria		
LPGB_12		Cor_12	Ucria		
LPGB_13		Cor_13	Ucria		
LPGB_14		Cor_14	Ucria		
LPGB_15		Cor_15	Ucria		
LPGB_16		Cor_16	Ucria		
LPGB_17		Cor_17	Ucria		
LPGB_18		Cor_18	Ucria		
LPGB_19		Cor_19	Ucria		
LPGB_20		Cor_20	Ucria		
LPGB_21		Cor_21	Ucria		
LPGB_22		Cor_22	Ucria		
LPGB_23		Cor_23	Ucria		
LPGB_24		Cor_24	Ucria		
LPGB_25		Cor_25	Ucria		
LPGB_26		Cor_26	Ucria		
LPGB_27		Cor_27	Ucria		
LPGB_28		Cor_28	Ucria		
LPGB_29		Cor_29	Ucria		
LPGB_30		Cor_30	Ucria		

* FC=Field Collection

**commercial and local varieties registred into Italian "National Register of Plant Variety"

Table S10. List of descriptors evaluated in 30 nuts for each Sicilian genotypes sampled in Nebrodi Mountains.

Descriptor	Method / Score scale*	
Nut: size	very small [1]	Example Varieties: Morell
	small [3]	Example Varieties: Negret, Tombul, Tonda Gentile delle Langhe
	medium [5]	Example Varieties: Segorbe, Tonda di Giffoni
	large [7]	Example Varieties: Fertile de Coutard, Merveille de Bollwiller
	very large [9]	Example Varieties: Apoldaer Zellernuss, Bergeri, Ennis
Nut shell colour	greenish yellow [1]	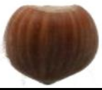
	light brown [2]	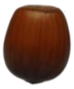
	brown [3]	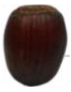
Nut – shape of cross section	elliptic [1]	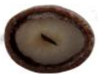
	circular [2]	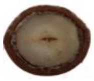
	triangular [3]	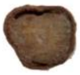
	rectangular [4]	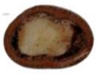
Nut: curvature of basal scar	concave [1]	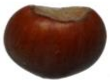
	even [2]	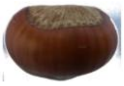
	convex [3]	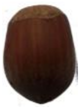

Descriptor	Method / Score scale*	
Nut shape in alteral view	circular [1]	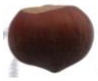
	triangular [2]	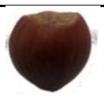
	ovate [3]	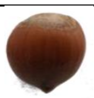
	oblong [4]	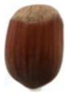
Nut: conspicuousness of stripes on shell	few [3]	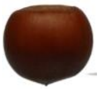
	medium [5]	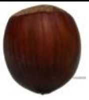
	many [7]	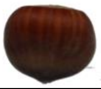
Nut: shape of apex	narrow acute [1]	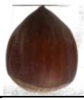
	broad acute [2]	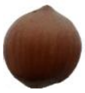
	obtuse [3]	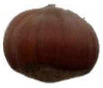
	truncate [4]	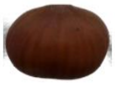

* in square brackets

Table S11. Summary of the 9 SSR markers,primer multiplex and co-loading sets used in the study.

SSr locus	Repeat motif	Primer sequences	Dye label	Multiplex set	Expected size	References
CAC-B020	GA)$_{19}$	F_GGGAAAATACTCCAAATCGCT<br>R_TCACCGAGCCGTCATAATC	FAM	2	275-289	[26]
CAC-B028	AG)$_{16}$	F_ATG GAC GAG GAA TAT TTC AGC<br>R_CCT GTT TCT CTT TGT TTT CGA G	HEX	1	255-278	
CAC-B105	(GA)$_{16}$	F_AAAGGAGCAAGCATGTTAGG<br>R_GTTTGTACGGATGATCCACTGA	FAM	3	153-159	[28]
CAT-B107	(CT)$_{14}$	F_TAG GTG CAC TTG ATG TGC TTT AC<br>R_AAC ACC ATA TTG AGT CTT TCA AAG C	HEX	1	114-147	
CAT-B502	(CT)$_{16}$GCTTTTC(CT)$_{5}$	F_CTC ATG ACT GCC CAT TTC TCG<br>R_AGG CAT GCA GGC TTC ACA C	FAM	1	185-205	
CAT-B501	(GA)$_{18}$	F_GAA ATT CAA TCA CAC CAA TAA AGC A<br>R_CCT CCC TTG TCC TCA TCA CTG	HEX	2	116-130	
CAT-B504	(CT)$_{18}$	F_CGCCATCTCCATTTCCCAAC<br>R_CGGAATGGTTTTCTGCTTCAG	FAM	4	161-185	
CAT-B505	(CT)$_{17}$CC(CT)$_{2}$	F_AGAGAACGACTTTGTATGACAAAGA<br>R_TTGAACCATTAAATACATCATGTGA	HEX	3	108-128	
CAT-B507	(GA)$_{1}$GC(GA)$_{2}$GC(GA)$_{14}$	F_CTAAGCTCACCAAGAGGAAGTTGAT<br>R_GCTTCTGGGTCTCCTGCTCA	HEX	3	180-198	
